# Nature’s cryptographic codebreaker*: in silico* decoding of apigenin’s triple defense against SARS-CoV-2

**DOI:** 10.3389/fmicb.2025.1708660

**Published:** 2025-12-16

**Authors:** Juanjuan Huang, Yabo Fang, Hongya Guan, Shoukui Hu

**Affiliations:** 1 Department of Clinical Laboratory, Zhengzhou Central Hospital Affiliated to Zhengzhou University, Zhengzhou, China; 2 China Unicom IoT Co., Ltd., Nanjing, China; 3 Department of Clinical Laboratory, Beijing Hospital of Traditional Chinese Medicine, Affiliated to Capital Medical University, Beijing, China

**Keywords:** apigenin, SARS-CoV-2, matrix factorization, deep learning, molecular modeling

## Abstract

**Introduction:**

The coronavirus disease 2019 (COVID-19) pandemic underscored the urgent need for broad-spectrum antiviral agents capable of targeting both viral proteins and host factors to mitigate disease severity. Apigenin has antiviral and anti-inflammatory properties. However, the potential of apigenin against SARS-CoV-2 remains insufficiently explored.

**Methods:**

In this study, the potential role of apigenin in the treatment of severe acute respiratory syndrome coronavirus 2 (SARS-CoV-2) and the underlying mechanism were explored using matrix factorization, deep learning, multiscale molecular modeling and network pharmacology.

**Results:**

The graph-based integrated Gaussian kernel similarity (GiGs) model predicted that apigenin might be a drug against SARS-CoV-2. The prediction of drug-target affinity using a convolution model with self-attention (CSatDTA) model revealed the potential binding affinity of apigenin with glucose-regulated protein 78 (GRP78) and heparan sulfate proteoglycan (HSPG). Molecular docking further validated strong binding to GRP78 (–8.198 kcal/mol) and moderate binding to HSPG (–5.6 kcal/mol), mediated by van der Waals forces and hydrogen bonds. Multiscale molecular modeling revealed that apigenin could bind to Non-structural protein 15 (Nsp15). Further, the network pharmacology analysis implied that apigenin might modulate the host inflammatory responses by potentially regulating the PI3K-Akt and HIF-1 signaling pathways and binding directly to protein kinase B (AKT1) and prostaglandin endoperoxide synthase 2 (PTGS2).

**Discussion:**

Computational profiling suggests apigenin exerts a multi-target mechanism against SARS-CoV-2, potentially disrupting viral entry, replication, and host inflammatory responses. The findings of this research outline a promising strategy and provide a rationale for developing novel natural product-based treatment methods for SARS-CoV-2.

## 1 Introduction

The coronavirus disease 2019 (COVID-19) pandemic was caused by severe acute respiratory syndrome coronavirus 2 (SARS-CoV-2) and spread across the globe repeatedly, posing a persistent threat to global health and economy (Chavda et al., 2022). Although vaccines and the approved antiviral drugs have changed the course of the epidemic, broad-spectrum antiviral drugs against the disease are still lacking owing to adverse reactions and drug resistance (Li et al., 2022; Stevens et al., 2022). Traditional drug discovery has long discovery cycles and high costs (Wang et al., 2021). Therefore, computational drug repositioning has become a promising alternative strategy to rapidly identify existing drugs for treating new diseases (Kumar et al., 2022). Advanced computational methods have been used to rapidly identify effective antiviral drugs, serving as a powerful strategy for treatment against SARS-CoV-2.

Antiviral and anti-inflammatory treatments are key to COVID-19 treatment (Kim et al., 2021). Targeting the host factors for viral entry, such as glucose-regulated protein 78 (GRP78) and heparan sulfate proteoglycan (HSPG), has emerged as an attractive therapeutic approach to prevent the initial infection (Gao et al., 2022; Ha et al., 2023). Concurrently, the inhibition of conserved viral enzymes serves as another pivotal approach; for example, Non-structural protein 15 (Nsp15), an endoribonuclease critical for viral replication, was investigated as a promising drug target for SARS-CoV-2 infection (Hu et al., 2022). In addition to direct antiviral strategies, mitigating the inflammatory response is equally critical. Overactivation of the host inflammatory pathways, such as the PI3K-Akt and HIF-1 signaling pathways, contributes to the generation of the cytokine storm (Huynh et al., 2025). Therefore, the development of a drug capable of simultaneously inhibiting viral entry and replication while mitigating the host inflammatory response represents a promising strategy against SARS-CoV-2.

Apigenin is a dietary flavonoid that is abundant in various vegetables, fruits, and plant-based beverages (Hostetler et al., 2017). Apigenin has attracted widespread attention because of its broad pharmacological properties, including anti-inflammatory, anti-cancer, and antioxidant properties (Patil et al., 2016; Cheng et al., 2021; Singh et al., 2025). Accumulating evidence has demonstrated that apigenin also exhibits antiviral effects against multiple viruses, such as the influenza virus, hepatitis C virus, enterovirus 71, and Zika virus (Liu et al., 2012; Lv et al., 2014; Sangeetha et al., 2020; Libin et al., 2024). In addition, research on its metabolite indirectly supports therapeutic potential. Apigenin-7-O-glucuronide, the main metabolite of apigenin, has been shown to have inhibitory activity against Rift Valley Fever Virus, which the binding energy is −7.3 kcal/mol (Dutt et al., 2025). However, the potential of apigenin in preventing SARS-CoV-2 infection remains insufficiently explored.

**FIGURE 1:**
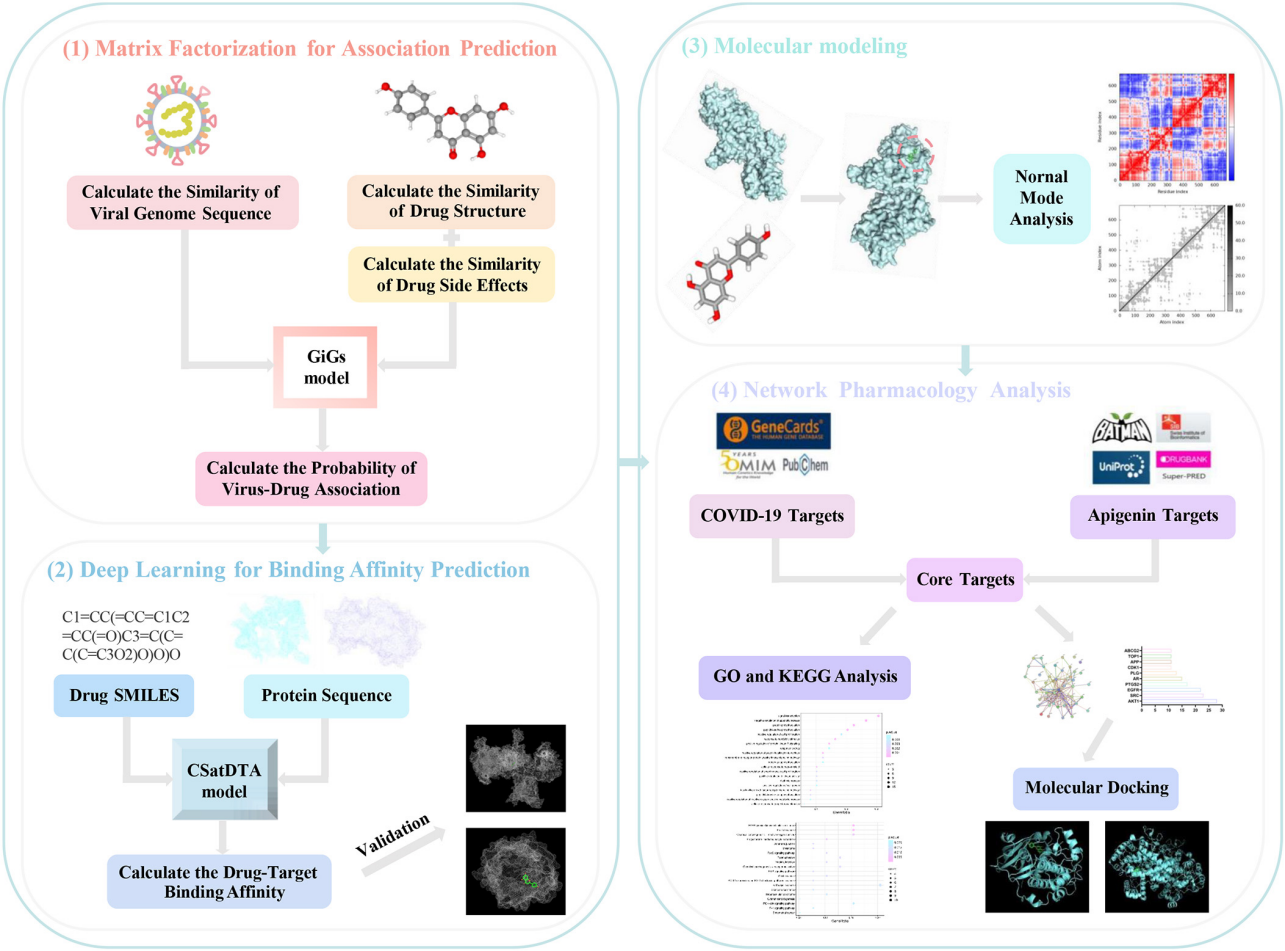
The flowchart of this study. The diagram outlines the key steps: (1) Construction and application of the GiGs model for virus-drug association probability prediction; (2) Construction and application of the CSatDTA model for drug-target binding affinity prediction; (3) Multiscale molecular modeling analysis of apigenin and Nsp15; (4) Network pharmacology analysis of apigenin against SARS-CoV-2.

In this study, we employed a multi-tiered computational strategy to systematically investigate the anti-SARS-CoV-2 potential of apigenin (Figure 1). First, the graph-based integrated Gaussian kernel (GiGs) algorithm was applied to predict the association probability between apigenin and SARS-CoV-2 (Jin et al., 2025). To delve into the mechanism, we then explored interactions with key viral targets across different life cycle stages. This involved predicting the binding affinity for viral entry proteins (GRP78 and HSPG) using a convolution-based self-attention (CSatDTA) model (Ghimire et al., 2022), validating these interactions through molecular docking. Moreover, multiscale molecular modeling was performed to explore the binding mechanism between apigenin and viral replication Nsp15. Finally, a network pharmacology analysis was conducted to identify the core targets and signaling pathways involved in the anti-SARS-CoV-2 effects of apigenin. This comprehensive computational strategy aims to explore the multitarget potential of a common dietary flavonoid against viral invasion, replication, and host inflammation, thereby proposing a hypothesis and establishing a potential AI-powered framework for rapid drug repositioning in global health emergencies.

## 2 Materials and methods

### 2.1 Prediction of virus-drug association using the GiGs algorithm

The GiGs algorithm was employed to predict the association probability between apigenin and SARS-CoV-2. The training data included 95 viruses, 175 drugs, and 933 confirmed VDAs. The known anti-SARS-CoV-2 compounds nirmatrelvir, baicalin and luteolin were included as positive controls. The optimized parameters for GiGs were determined as follows: weight parameter (α) = 1, regularization parameters (λ_1_, λ_2_, λ_3_) = (16, 0.125, 16), and latent feature vector (*k*) = 70. The SMILES strings of apigenin, nirmatrelvir, baicalin, and luteolin were retrieved from the PubChem database. Viral genome sequence similarity was computed using MAFFT. Drug chemical structure and side effect similarities were calculated using SIMCOMP and Jaccard scores, respectively. These similarities were integrated using the GIPK method. Finally, the association probability for each virus-drug pair was predicted based on a similarity-constrained weight graph regularization matrix factorization framework.

### 2.2 Prediction of drug-target binding affinity using the CSatDTA algorithm

The binding affinity of apigenin with GRP78 and HSPG was predicted using the CSatDTA model. The CSatDTA model was trained with an initial learning rate of 0.001, a batch size of 64, and the Adadelta optimizer. The network architecture consisted of two convolutional neural network (CNN) layers and two attention layers. For the attention mechanisms, the SMILES and protein inputs utilized 4 and 10 attention heads, with 2 and 5 filters for keys and values, respectively. The SMILES string of apigenin was retrieved from the PubChem database. The amino acid sequences of GRP78 and HSPG were acquired from NCBI database. The SMILES of apigenin and the amino acid sequence of these proteins were input into the CSatDTA model, which generated a predicted binding affinity score to estimate the strength of the interaction.

### 2.3 Molecular docking

The two-dimensional (2D) structure of apigenin was retrieved from the PubChem database (Kim et al., 2025). The three-dimensional (3D) crystal structures of the target proteins were acquired from the RCSB PDB database (Burley et al., 2021), including GRP78 (PDB ID: 5E84), HSPG (PDB ID: 5E9C), Nsp15 (PDB ID: 6w01), AKT1 (PDB ID: 3QKL), and PTGS2 (PDB ID: 5f19). And we removed water, added hydrogen, and calculated the charge from these protein structures. AutoDockTools was used to convert these structures into PDBQT format (Morris et al., 2009). The docking box information is shown in Supplementary Table 1. For GRP78, the size of gird box was set to 180.495 × 180.495 × 180.495, and the coordinates of center were set to center_x = 43.093, center_y = 45.547 and center_z = −39.746. For HSPG, the size of gird box was set to 66.314 × 66.314 × 66.314, and the coordinates of center were set to center_x = −13.528, center_y = 13.240 and center_z = 62.862. For Nsp15, the size of gird box was set to 62.488 × 62.488 × 62.488, and the coordinates of center were set to center_x = −54.398, center_y = 54.254 and center_z = 23.211. For AKT1, the size of gird box was set to 25.008 × 25.008 × 25.008, and the coordinates of center were set to center_x = −7.455, center_y = 7.326 and center_z = 22.827. For PTGS2, the size of gird box was set to 65.192 × 65.192 × 65.192, and the coordinates of center were set to center_x = 22.499, center_y = 41.040 and center_z = 59.075. And all docking run options were kept at their default values. Subsequently, molecular docking was performed using AutoDock Vina to calculate the binding energies and study binding sites (Eberhardt et al., 2021). After docking, the model with the lowest binding energy was selected to visualized the 3D and 2D interactions by PyMOL and Discovery Studio software (Lill and Danielson, 2011).

### 2.4 Normal mode analysis

Normal mode analysis (NMA) is a computer simulation method, which is widely used to evaluate the stability of protein-ligand complexes. The iMODS server,^1^ as an effective and user-friendly tool, was employed to perform NMA for the Nsp15-apigenin complex. The PDB file of the complex needed to be uploaded, and the server would measure its stability by providing deformability, B-factor, eigenvalues, variance, covariance map, and elastic network.

### 2.5 Identification of intersecting targets for apigenin and COVID-19

To identify the therapeutic targets of apigenin against COVID-19, the intersecting targets between the drug and the disease were systematically retrieved. COVID-19-related targets were obtained from PubChem, GeneCards, and OMIM databases. Potential targets of apigenin were acquired from multiple databases, including Batman, Drugbank, Swiss Target Prediction, SuperPred, and Uniprot. The intersecting targets between apigenin and COVID-19 were visualized using a Venn diagram.

### 2.6 Construction of protein-protein interaction network

To explore the interactions among the intersecting targets, a PPI network was constructed. The analysis was conducted using the STRING database (version 12.0). The intersecting targets were imported into STRING, with “*Homo sapiens*” set as the organism and a minimum interaction confidence score threshold of > 0.4. The resulting PPI network was then analyzed using Cytoscape software (version 3.9.1). And top 10 hub targets were identified based on the degree algorithm, which prioritizes nodes with the highest number of interactions within the network.

### 2.7 GO and KEGG enrichment analysis

To elucidate the biological functions and pathways modulated by the intersecting targets, GO and KEGG enrichment analyses were performed using the DAVID platform. The list of intersecting targets was submitted to DAVID. *P*-value and FDR < 0.05 were set for enrichment. The results for biological processes (BP), cellular components (CC), molecular functions (MF), and KEGG pathways were visualized using bubble plots to represent the significance of enrichment. Additionally, the “pathview” package was employed to generate pathway maps for the targets.

## 3 Results

### 3.1 GiGs model predicted apigenin as a potential anti-SARS-CoV-2 drug

**FIGURE 2:**
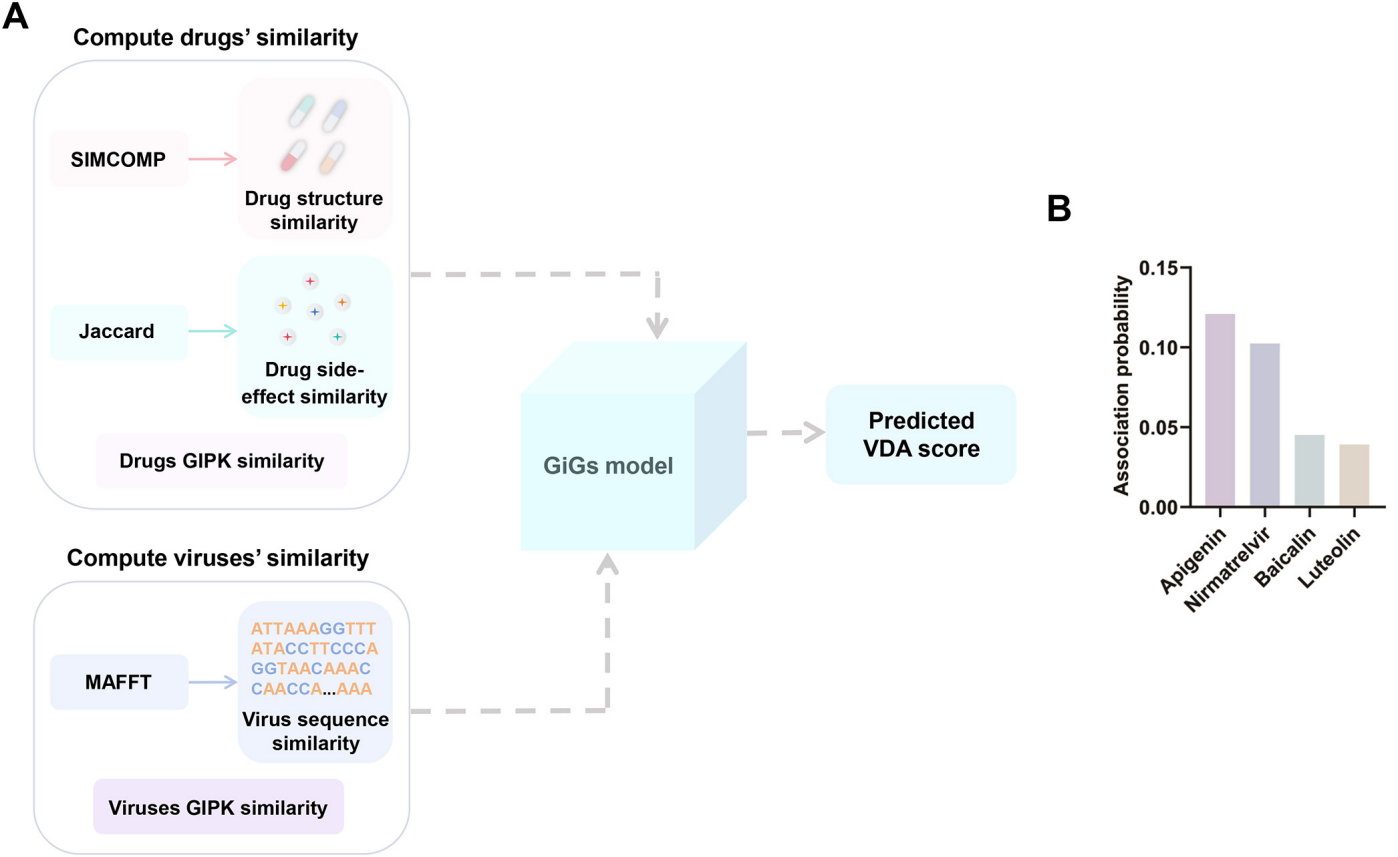
(A) Framework of the GiGs model. The model is trained on the DrugVirus dataset, with key hyperparameters set to: α = 1, λ_1_ = 16, λ_2_ = 0.125, λ_3_ = 16, *k* = 70 (Jin et al., 2025). (B) The association probability of SARS-CoV-2 with apigenin and control drugs (nirmatrelvir, baicalin, luteolin) predicted by GiGs model. The association probability ranges from 0 to 1, with 1 indicating the highest probability of association.

In order to assess the therapeutic potential of apigenin against SARS-CoV-2, the GiGs computational model was adopted to quantify the virus-drug association probabilities (Figure 2A; Jin et al., 2025). Nirmatrelvir has been clinically approved for treating SARS-CoV-2. Baicalin has also shown significant antiviral activity against SARS-CoV-2. Luteolin is a top-ranked flavonoid that has been reported against the SARS-CoV-2 and emerging Omicron variants. Therefore, these three drugs were used in this study as positive control drugs for comparison (Zhao et al., 2022; Noske et al., 2023; Kumar et al., 2025). As shown in Figure 2B, Supplementary Table 2, the GiGs model predicted an association probability of 0.120969785253999 for apigenin with SARS-CoV-2. This value notably exceeded the corresponding values noted for both the control drugs nirmatrelvir (0.102460103240095), baicalin (0.0451910863413688), and luteolin (0.0391282038177052). The superior correlation probability observed through the computational analysis provides strong preliminary evidence that apigenin is a potential drug for treating SARS-CoV-2 infection.

### 3.2 Uncovering apigenin binding to GRP78 and HSPG through integrated computational modeling

**FIGURE 3:**
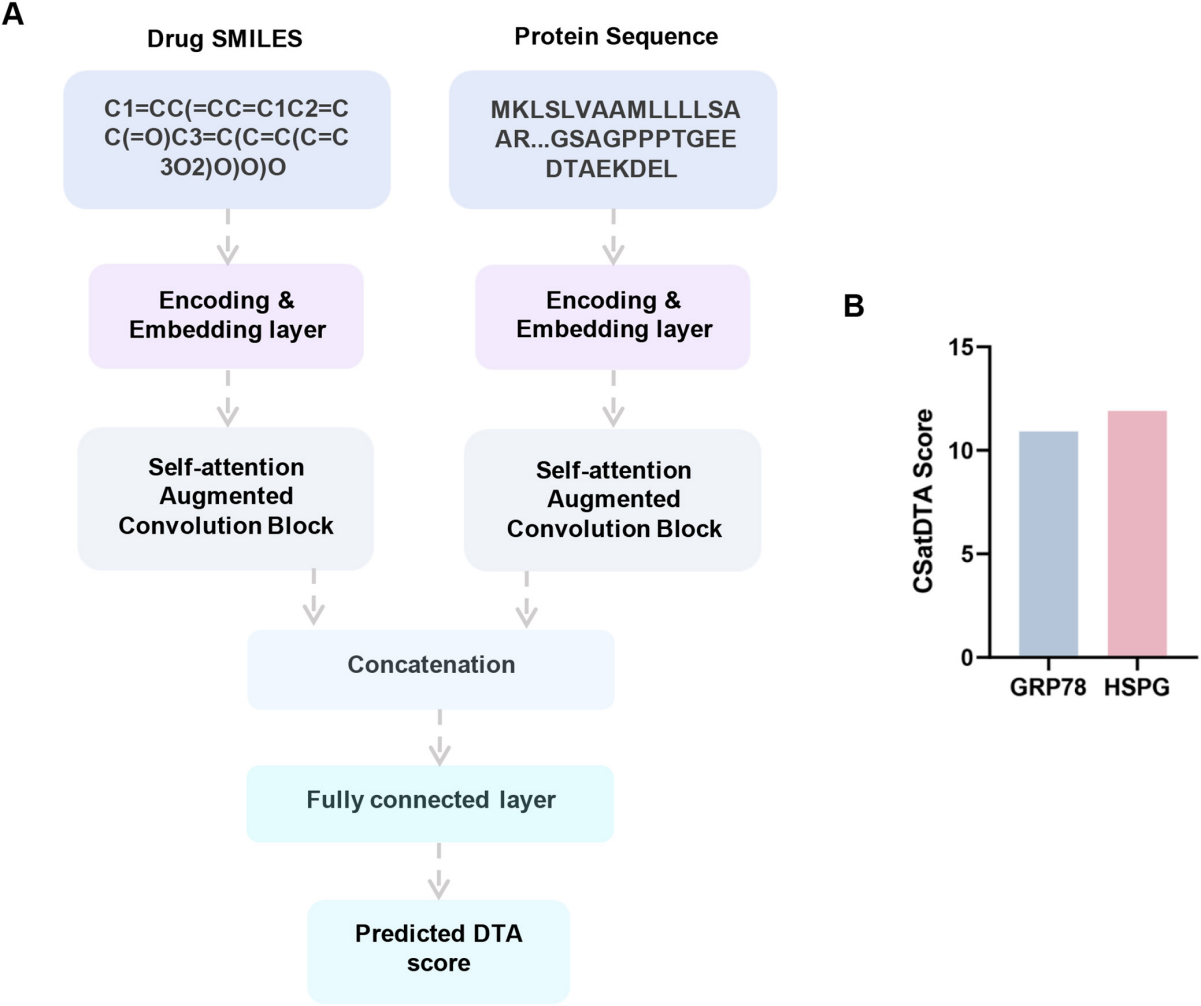
(A) Framework of the CSatDTA model, a convolution model with self-attention model for drug-target affinity prediction, trained on the KIBA dataset at an initial learning rate of 0.001 (Ghimire et al., 2022). (B) The binding affinity scores of apigenin with GRP78 and HSPG predicted by CSatDTA model. Higher scores indicate stronger binding.

GRP78 and HSPG have been identified as important host factor and cofactor promoting the entry of SARS-CoV-2. Therefore, to investigate whether apigenin can target these proteins, the CSatDTA model was adopted to predict their binding affinities (Figure 3A; Ghimire et al., 2022). The SMILES of apigenin and the amino acid sequences of the GRP78 and HSPG proteins were input into the model, and the predicted DTA score was the output. As shown in Figure 3B and Supplementary Table 3, apigenin presented potential binding affinities with GRP78 (score: 10.915016) and HSPG (score: 11.910124), indicating that it can target these host proteins.

**FIGURE 4:**
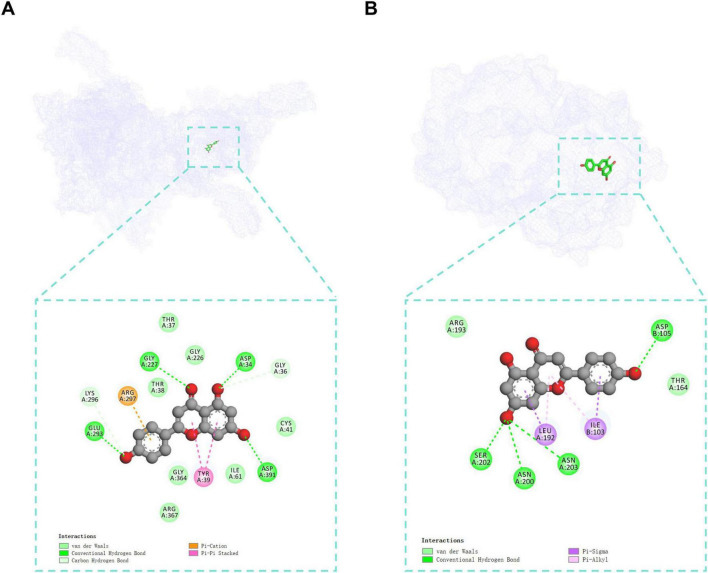
Molecular interactions of GRP78 (PDB ID: 5E84) and HSPG (PDB ID: 5E9C) with apigenin. (A) GRP78-apigenin. (B) HSPG-apigenin. The purple mesh structures represent the proteins, and the green rods represent apigenin.

Next, to elucidate the structural basis of these interactions, molecular docking was performed. Apigenin exhibited strong binding affinity for GRP78 (–8.198 kcal/mol), which was attributed to van der Waals forces (THR37, GLY226, THR38, GLY364, ARG367, ILE61, and CYS41), hydrogen bonds (GLY227, GLU293, ASP391, ASP34, LYS296 and GLY36), Pi-Cation (ARG297), and Pi-Pi stacked (TYR39) (Figure 4A and Supplementary Table 4). Moreover, the binding affinity of HSPG-apigenin was –5.6 kcal/mol, which was attributed to van der Waals forces (ARG193 and THR164), hydrogen bonds (SER202, ASN200, ASN203, and ASP105), and hydrophobic interactions (LEU192 and ILE103) (Figure 4B and Supplementary Table 4). Together, our predictive algorithm and molecular docking results suggested that apigenin may interfere with viral entry by binding to GRP78 and HSPG.

### 3.3 Multiscale molecular modeling predicts that apigenin binds to the SARS-CoV-2 Nsp15 protein

**FIGURE 5:**
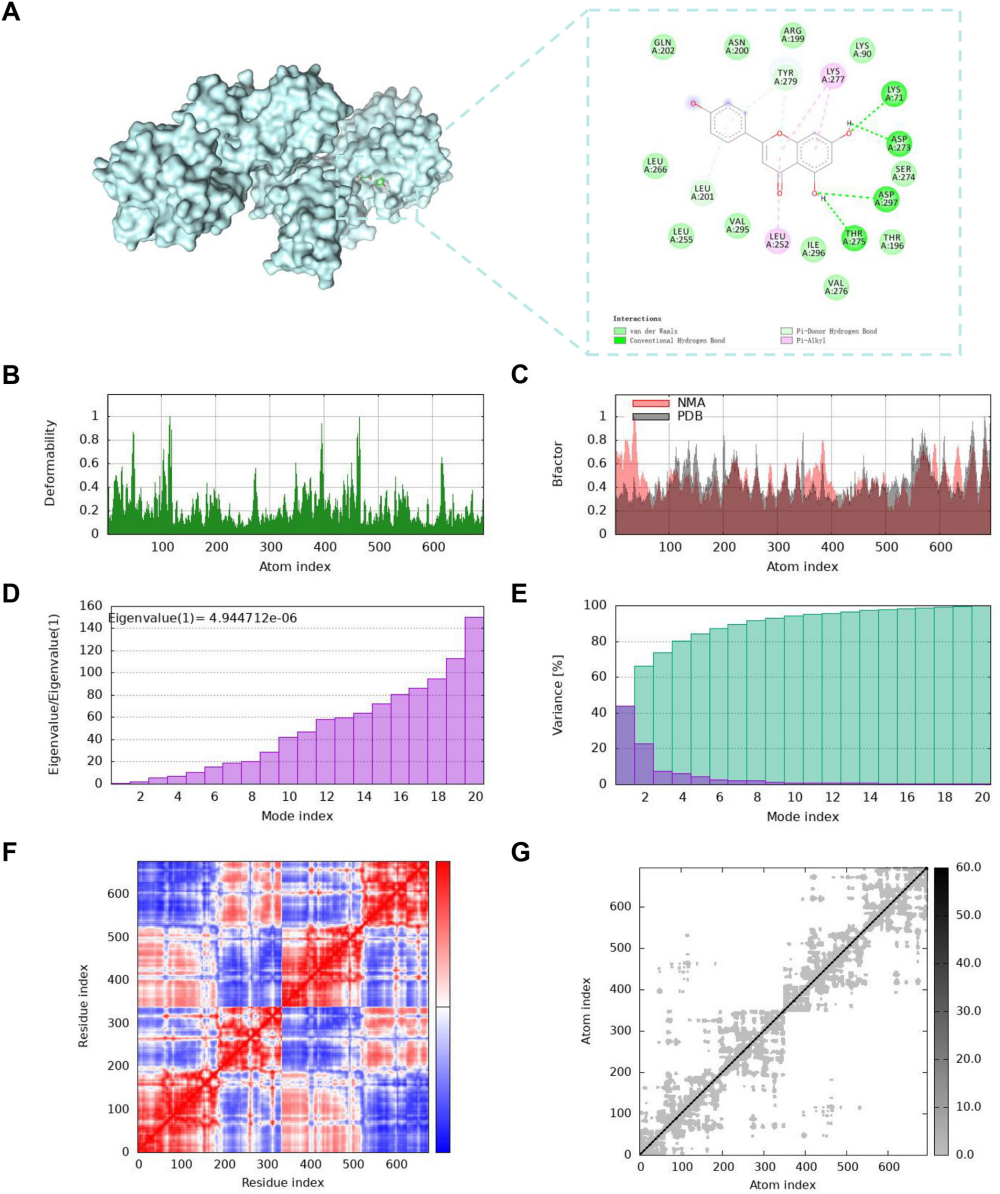
Molecular docking and normal mode analysis of apigenin and Nsp15. (A) The docking mode and 2D interaction between apigenin and Nsp15 (PDB ID: 6w01). The blue surface structures represent Nsp15 protein, and the green rods represent apigenin. (B) Deformability. (C) B-factor. (D) Eigenvalues. (E) Variance. (F) Covariance map. (G) Elastic network.

Nsp15 is crucial for virus replication and is therefore an attractive drug target for treating SARS-CoV-2. In this study, Nsp15 was studied for its potential interaction with apigenin using molecular docking. The optimal docking model of Nsp15-apigenin is shown in Figure 5A and Supplementary Table 4. The binding affinity of Nsp15-apigenin was −8.177 kcal/mol, and this interaction was attributed to van der Waals forces (GLN202, LEU266, LEU255, VAL295, ILE296, VAL276, THR196, SER274, LYS90, ARG199, and ASN200), hydrogen bonds (THR275, ASP297, ASP273, LYS71, LEU201, and TYR279), and hydrophobic interactions (LEU252 and LYS277). In addition, the stability of Nsp15-apigenin was determined using the normal mode analysis. The deformability of Nsp15-apigenin is shown in Figure 5B, and the pivots indicated a high deformation area. Figure 5C shows the B factor of Nsp15-apigenin between NMA mobility and the PDB field. The lower the eigenvalues are, the easier it is to deform the structure. The eigenvalues of the complex was 4.944712e-06, indicating that the complex was stable (Figure 5D). The variance of Nsp15-apigenin is shown in Figure 5E, with purple indicating individual variance and green representing cumulative variance. The covariance map revealed correlations, a lack of correlation, and an inverse correlation between amino acid residues (Figure 5F). The elastic network of Nsp15-apigenin is shown in Figure 5G, and each point in the figure represents a spring between atomic pairs, with darker gray indicating harder springs. These findings suggested that apigenin could potentially interfere with viral replication by binding to Nsp15.

### 3.4 Systems pharmacology analysis of apigenin against SARS-CoV-2

**FIGURE 6:**
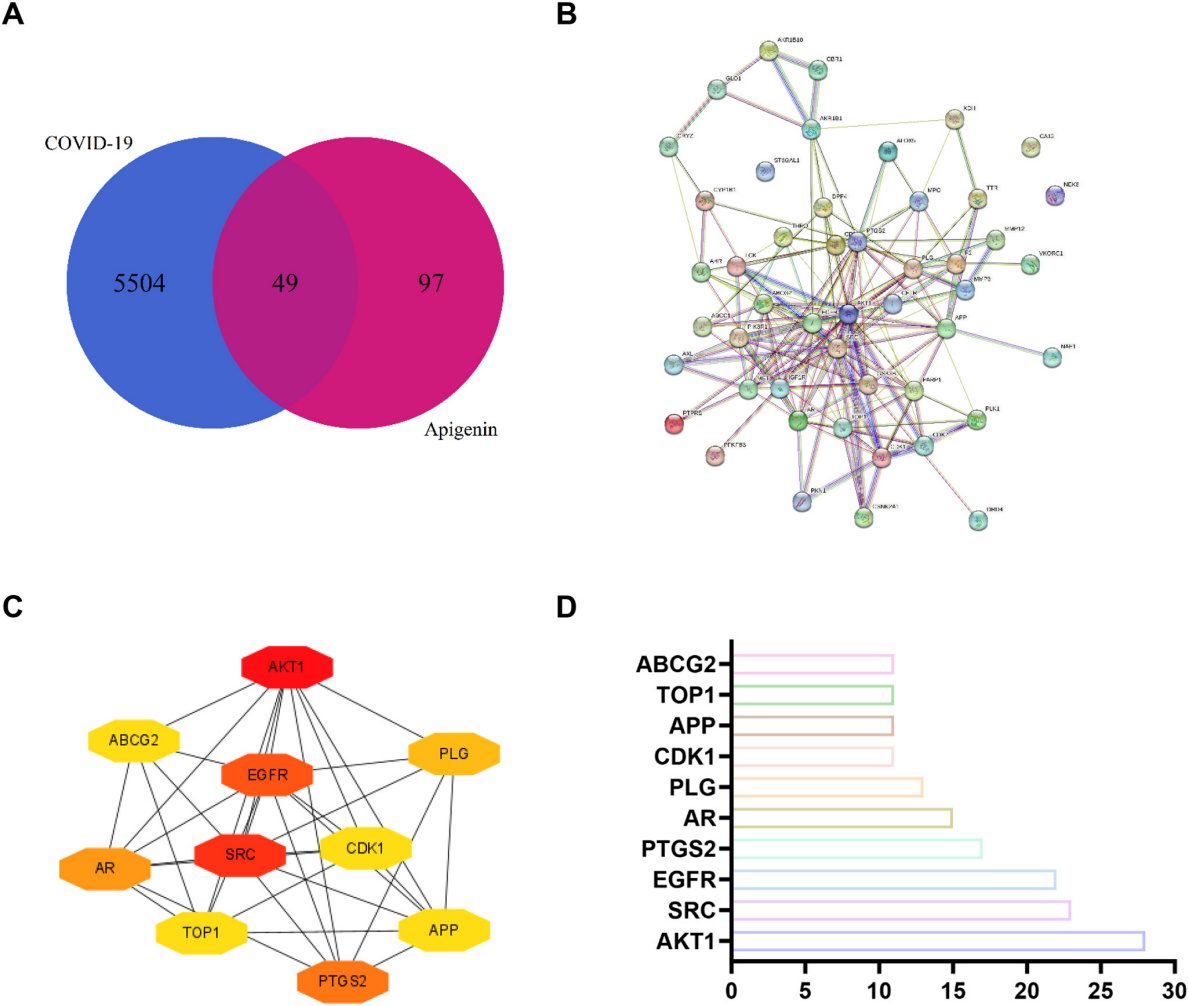
Intersecting gene analysis of SARS-CoV-2 and apigenin. (A) The Venn diagram of intersecting genes. Note: purple areas denote apigenin targets, and blue areas denote SARS-CoV-2 targets. (B) PPI network of intersecting genes. (C) Top 10 hub genes ranked by degree algorithm. (D) Bar chart of degree value for hub genes.

In order to further investigate the mechanism by which apigenin affects SARS-CoV-2, a network pharmacology approach was adopted. A total of 146 targets of apigenin and 5,553 targets of SARS-CoV-2 were obtained from multiple databases. The constructed Venn diagram revealed 49 intersecting targets, which were potential therapeutic targets of apigenin against SARS-CoV-2 (Figure 6A). The PPI network of these intersecting targets is shown in Figure 6B, the network consists of 48 nodes and 182 edges after setting the confidence level > 0.4. The top 10 hub genes identified through the degree algorithm included AKT1, SRC, EGFR, PTGS2, AR, PLG, CDK1, APP, TOP1, and ABCG2 (Figures 6C,D). Nodes in the network are represented by different colors, ranging from yellow to red. The redder the color, the more likely it is to be the core target. Supplementary Table 5 listed detailed information on the top 10 hub genes.

**FIGURE 7:**
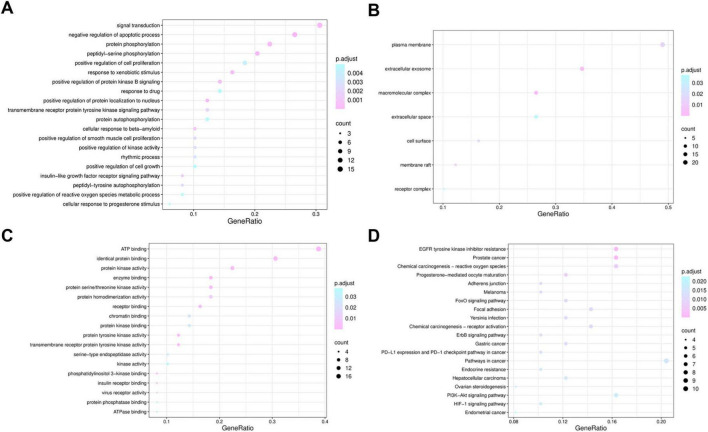
GO and KEGG enrichment analysis of intersecting targets of apigenin against SARS-CoV-2. (A) Top 20 BP terms. (B,C) CC and MF terms. (D) Top 20 KEGG signaling pathways. The color of the node corresponds to the significance level of the adjust *p*-value, and its size represents the number of genes enriched in the pathway.

GO and KEGG enrichment analyses were subsequently conducted to decipher the biological functions of the 49 intersecting targets. Terms with an FDR < 0.05 were considered statistically significant. In the resultant bubble plots, the color of each node corresponds to the statistical significance level, and the node size represents the number of genes enriched in the respective term or pathway. GO analysis revealed 36 BP, 7 CC, and 18 MF terms that were significantly enriched (Supplementary Table 6). Among these, the most significantly enriched BP terms included peptidyl-serine phosphorylation, negative regulation of apoptotic process, protein phosphorylation, signal transduction, and positive regulation of reactive oxygen species metabolic process (Figure 7A). The representative CC terms included membrane raft and cell surface (Figure 7B). The representative MF terms included protein kinase activity, phosphatidylinositol 3-kinase binding, and virus receptor activity (Figure 7C). The KEGG pathway analysis revealed 37 significantly enriched pathways (Supplementary Table 7). Bubble plots of the top 20 pathways are shown in Figure 7D, and these mainly involved the PI3K-Akt signaling pathway and the HIF-1 signaling pathway. The relevant targets in the key inflammatory pathways are shown in Supplementary Figures 1, 2.

### 3.5 Molecular docking validation of apigenin with hub targets

**FIGURE 8:**
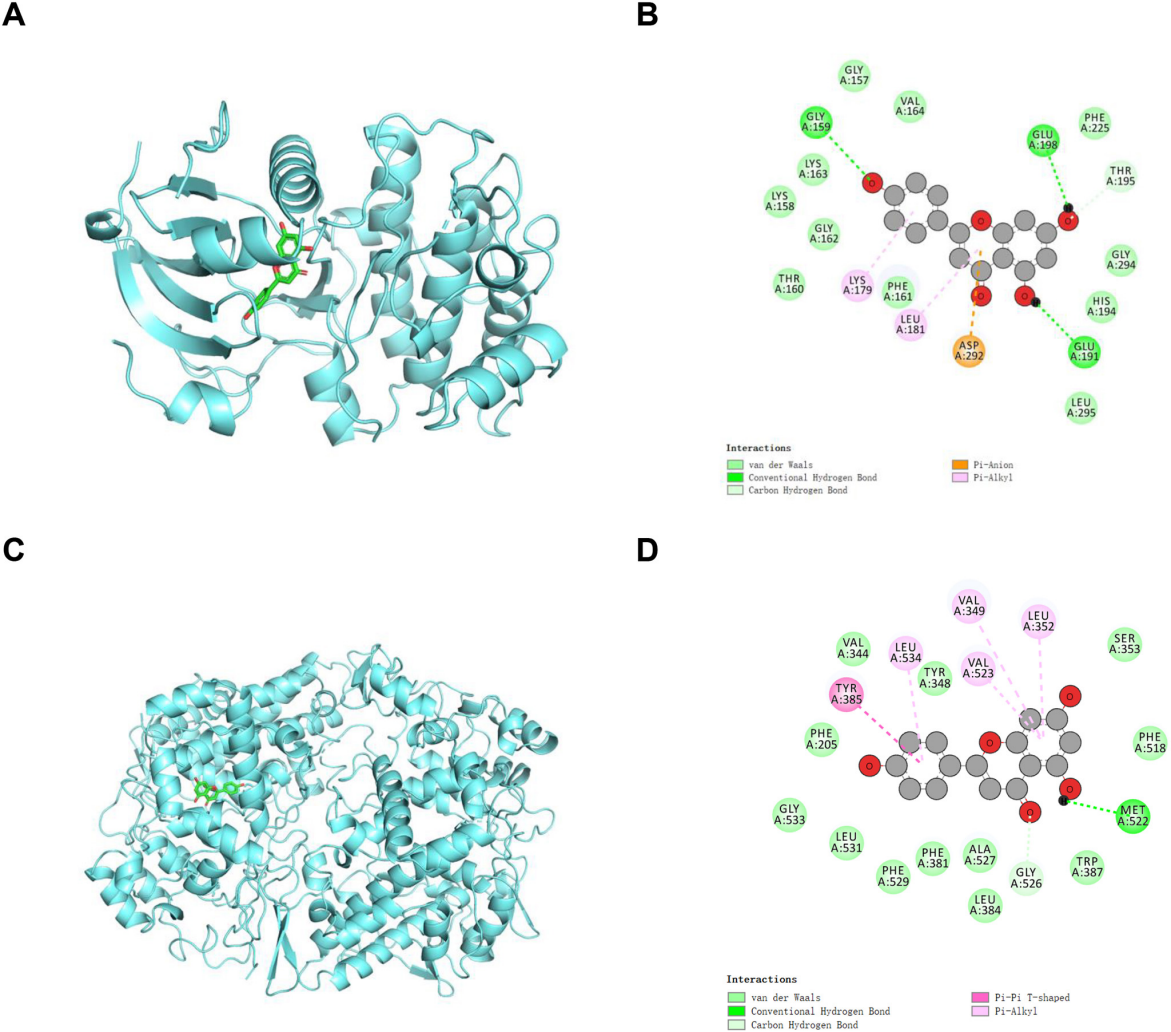
Molecular docking of apigenin and hub targets. (A,B) 3D docking model and 2D interaction diagram of apigenin with AKT1 (PDB ID: 3QKL). (C,D) 3D docking model and 2D interaction diagram of apigenin with PTGS2 (PDB ID: 5f19). The blue spirals show the protein structures, and the green rods show the apigenin.

Given the central roles of AKT1 and PTGS2 in inflammatory responses, molecular docking was performed in this study to validate the direct binding of AKT1 and PTGS2 with apigenin and explore their specific molecular interactions. The blue spiral represents proteins, whereas the green rod-shaped structure represents drugs. As shown in Figures 8A,B and Supplementary Table 4, the binding free energy of AKT1-apigenin was –9.41 kcal/mol, which was attributed to van der Waals forces with the VAL164, GLY157, LYS163, LYS158, GLY162, THR160, PHE161, LEU295, HIS194, GLY294, and PHE225 residues, hydrogen bonds with the GLY159, GLU191, THR195, and GLU198 residues, Pi-Anion interactions with the ASP292 residues, and Pi-Alkyl interactions with the LYS179 and LEU181 residues. The optimal docking model of PTGS2-apignein (–8.091 kcal/mol) is shown in Figure 8C, and it was attributed to van der Waals forces with the TYR348, VAL344, PHE205, GLY533, LEU531, PHE529, PHE381, ALA527, LEU384, TRP387, PHE518, and SER353 residues, hydrogen bonds with the GLY526 and MET522 residues, Pi-Pi T-shaped interactions with TYR385 residue, and Pi-Alkyl with the LEU534, VAL523, VAL349, and LEU352 residues (Figure 8D and Supplementary Table 4). These findings suggested that apigenin might exert its anti-inflammatory effect by targeting AKT1 and PTGS2.

## 4 Discussion

In order to address the urgent need for effective therapeutics against SARS-CoV-2, this study adopted an integrative computational framework that combines artificial intelligence, multiscale molecular modeling, and network pharmacology to systematically investigate the effects and mechanisms of apigenin against SARS-CoV-2. Computational drug repositioning plays an important role in the identification of effective drugs against SARS-CoV-2 (Dotolo et al., 2021). A deep learning ensemble model named DLEVDA has been proposed for use in the prediction of novel virus-drug associations, prioritizing the potential efficacy of drugs against SARS-CoV-2 (Deepthi et al., 2021). Another deep learning method named VDA-DLCMNMF is proposed for predicting potential drugs for treating novel viruses, providing further insights into the reuse of existing drugs for SARS-CoV-2 (Su et al., 2022). The GiGs algorithm was applied to prioritize the potential therapeutic effects, which predicted the association between apigenin and SARS-CoV-2. This initial prediction justified a deeper investigation into its potential mechanisms.

Inhibiting multiple targets has greater therapeutic potential for the treatment of COVID-19. GRP78 and HSPG are two important molecules for SARS-CoV-2 for its entry into cells and can be used as targets against this virus. A previous study showed that the celecoxib derivative AR12 could inhibit the expression of GRP78, enabling the treatment of SARS-CoV-2 (Rayner et al., 2020). Lactoferrin exerts antiviral activity against SARS-CoV-2 by targeting HSPG (Hu et al., 2021). In addition, Nsp15 is crucial for virus replication and is considered a drug target for SARS-CoV-2. Curcumin, a natural compound derived from turmeric, can strongly bind to the Nsp15 protein to inhibit virus replication (Kumar et al., 2021). Similarly, the extensively studied flavonoids EGCG and baicalin inhibit the Nsp15 with IC_50_ values of 1.62 ± 0.36 μM and 7.98 ± 1.46 μM, respectively (Hong et al., 2021). This finding establishes a clear precedent for flavonoid-based Nsp15 inhibition. We have established credible binding to GRP78, HSPG, and Nsp15 through cross-verification (including AI-docking concordance and NMA), and thus we propose that apigenin could exert its anti-SARS-CoV-2 effects by interfering with these pivotal proteins. This proposition is strongly supported by experimental findings demonstrating that apigenin inhibits SARS-CoV-2 replication *in vitro* with an IC50 of 5.11 ± 0.26 μM, a potency comparable to that of the widely studied flavonoid luteolin (IC_50_ = 5.92 ± 0.30 μM) (Chaves et al., 2022).

Our network pharmacology analysis demonstrated that mitigating the hyperinflammatory response is a potential mechanism underlying the therapeutic effect of apigenin against COVID-19, potentially implemented through the modulation of the PI3K-Akt and HIF-1 signaling pathways. This finding aligns with the existing literature, which reports that the PI3K-Akt signaling pathway regulates the activation of inflammatory cells, whereas the HIF-1 signaling pathway is associated with SARS-CoV-2-induced cytokine storms and inflammation (Serebrovska et al., 2020; Feng et al., 2022). The experimental finding that apigenin reduces TNF-α in SARS-CoV-2-infected Calu-3 cells provides direct phenotypic support for its role in mitigating inflammation (Chaves et al., 2022). The network pharmacology and molecular docking analyses conducted in this study pinpointed AKT1 and PTGS2 as hub targets within these networks. AKT1 is a key participant in COVID-19-related immune inflammation, while PTGS2 inhibition reportedly regulates the excessive inflammatory response of COVID-19 (Xia et al., 2020; Mallah et al., 2024). The concerted targeting of both AKT1 and PTGS2 by apigenin provides a plausible and multifaceted strategy to disrupt the inflammatory cascade, thereby offering a predictive rationale for positioning apigenin as a promising multitarget agent. In the future, we will involve performing molecular dynamics simulations to gain deeper insights into the stability and energetics of the binding interactions. Furthermore, we will carry out validation work including *in vitro* and *in vivo* experiments to ultimately confirm the therapeutic effect of apigenin against SARS-CoV-2.

## 5 Conclusion

In conclusion, this integrative study provides computational evidence suggesting that apigenin may offer a multi-pronged defense against SARS-CoV-2 by targeting viral entry, viral replication, and the host inflammatory response. The predicted mechanism underlying this effect is as follows: (i) it may disrupt virus entry by binding to GRP78 and HSPG; (ii) it could inhibit viral replication by binding to Nsp15; and (iii) it may modulate host inflammatory responses by regulating the PI3K-Akt and HIF-1 signaling pathways and targeting AKT1 and PTGS2. These findings suggest that apigenin employs a potential dual antiviral strategy that simultaneously targets the viral life cycle and the host inflammatory response, supporting its profile as a promising candidate for the further investigation as a multimodal treatment of SARS-CoV-2. The present integrated computational study thus provides a theoretical basis and a framework for further experimental validation on natural flavonoids.

## Data Availability

The raw data supporting the conclusions of this article will be made available by the authors, without undue reservation.

## Footnotes

1
http://imods.chaconlab.org/
