# Supplementary material for: Nature’s cryptographic codebreaker: in silico decoding of apigenin’s triple defense against SARS-CoV-2
Source: Front Microbiol. 2025 Dec 16;16:1708660. doi: 10.3389/fmicb.2025.1708660 (PMC12748202; doi:10.3389/fmicb.2025.1708660)
Supplement: Supplementary file 1 [file Data_Sheet_1.docx]

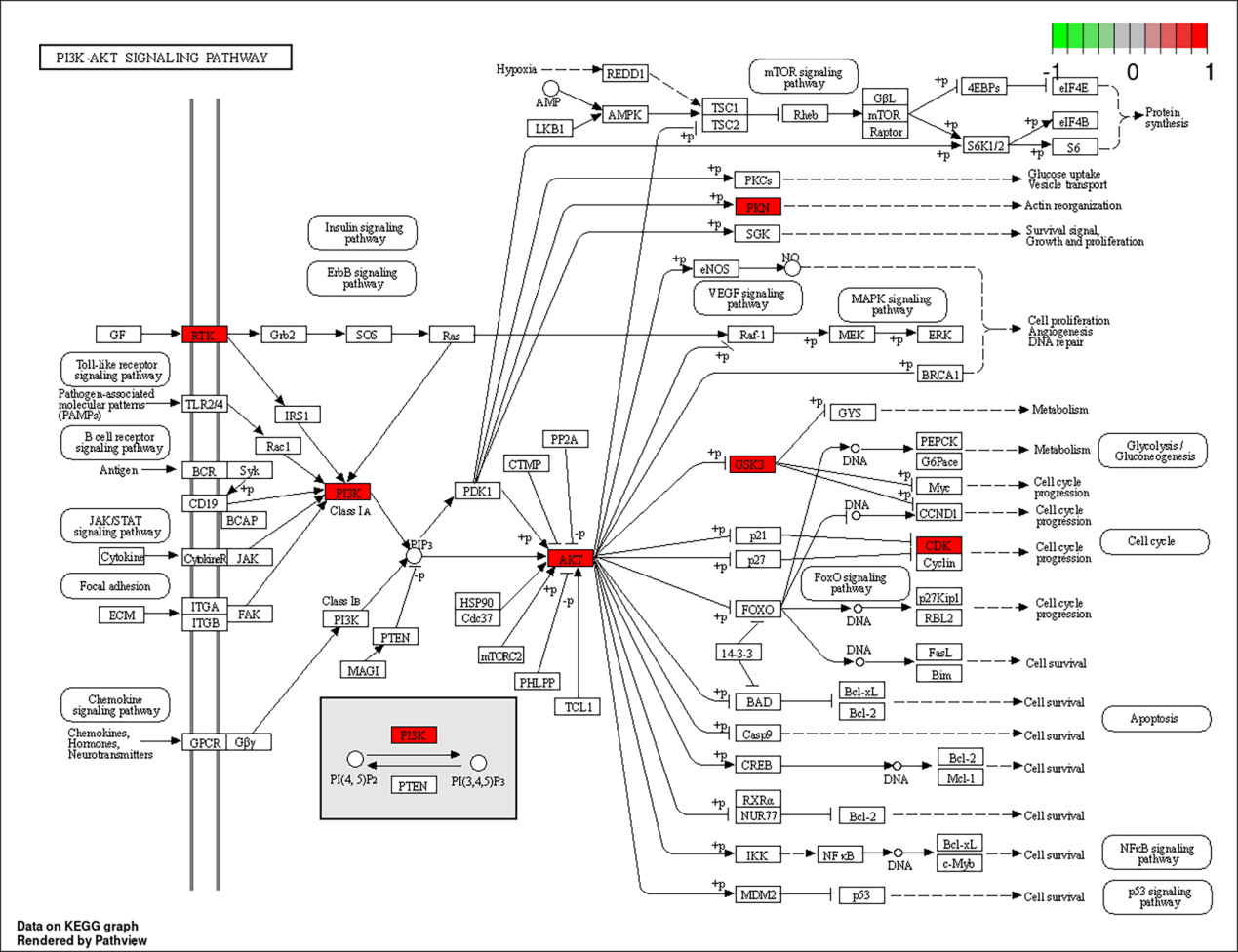


**FIGURE S1.** PI3K-Akt signaling pathway enrichment map. The red part denotes the targets of apigenin.


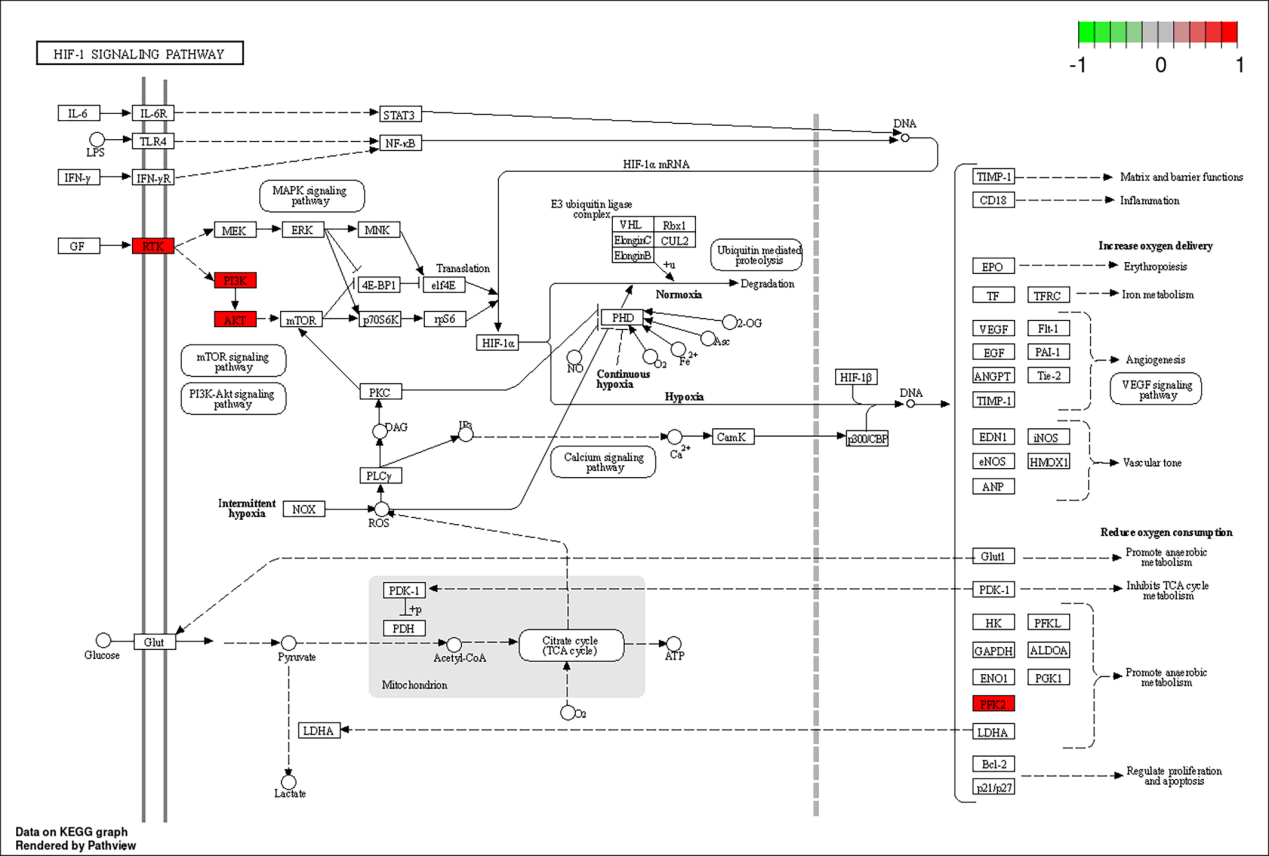


**FIGURE S2.** HIF-1 signaling pathway enrichment map. The red part denotes the targets of apigenin.
